# Positive Impact of Nutritional Interventions on Serum Symmetric Dimethylarginine and Creatinine Concentrations in Client-Owned Geriatric Cats

**DOI:** 10.1371/journal.pone.0153654

**Published:** 2016-04-14

**Authors:** Jean A. Hall, Jennifer MacLeay, Maha Yerramilli, Edward Obare, Murthy Yerramilli, Heidi Schiefelbein, Inke Paetau-Robinson, Dennis E. Jewell

**Affiliations:** 1 Department of Biomedical Sciences, College of Veterinary Medicine, Oregon State University, Corvallis, Oregon, United States of America; 2 Pet Nutrition Center, Hill's Pet Nutrition, Inc, Topeka, Kansas, United States of America; 3 IDEXX Laboratories, Inc., One IDEXX Drive, Westbrook, Maine, United States of America; Hospital Universitario de La Princesa, SPAIN

## Abstract

A prospective study was conducted in client-owned geriatric cats to evaluate the short- term effects of a test food on serum symmetric dimethylarginine (SDMA) and creatinine (Cr) concentrations. Test food contained functional lipids (fish oil), antioxidants (vitamins C and E), L-carnitine, botanicals (vegetables), highly bioavailable protein, and amino acid supplements. Cats (n = 80) were fed either test food or owner’s-choice foods (non-nutritionally controlled cohort). Cats were included based on age (≥ 9 years), indoor only, neutered, and free of chronic disease. At baseline, all cats had serum Cr concentrations within the reference interval. Renal function biomarkers and urinalysis results at baseline and after consuming test food or owner’s-choice foods for 3 and 6 months were evaluated. Cats consuming test food showed significant decreases in serum Cr and BUN concentrations across time. Overall, cats consuming owner’s-choice foods showed significant increases in serum SDMA concentrations at 3 and 6 months compared with baseline (*P* ≤ 0.05), whereas in cats consuming test food serum SDMA concentrations did not change. At baseline or during the 6-month feeding trial, 23 (28.8%) cats had increased serum SDMA, but normal serum Cr consistent with IRIS Stage 1 chronic kidney disease. This included 6 cats fed test food and 17 cats fed owner’s-choice foods. In the 6 cats fed test food, serum SDMA decreased in 3 cats and remained stable in 1 cat, whereas in the 17 cats fed owner’s-choice foods, serum SDMA increased in 13 cats and decreased or remained stable in 4 cats. The increase in serum SDMA concentration was significant (*P* = 0.02) only for cats fed owner’s-choice foods. These results suggest that nonazotemic cats with elevated serum SDMA (early renal insufficiency) when fed a food designed to promote healthy aging are more likely to demonstrate stable renal function compared with cats fed owner’s-choice foods. Cats fed owner’s-choice foods were more likely to demonstrate progressive renal insufficiency.

## Introduction

Early recognition of chronic kidney disease (CKD) is important for knowing when to initiate renoprotective interventions that may slow its progression, e.g., dietary modifications [1, 2]. Serum concentrations of symmetric dimethylarginine (SDMA) have been shown to detect CKD in cats on average 17.0 months before serum creatinine (Cr) concentration increased above the reference interval [3]. Therefore, serum SDMA is useful as a renal biomarker for identifying early compromise in renal function compared with serum Cr.

Symmetric dimethylarginine is produced when nitrogen molecules on arginine containing polypeptides are postranslationally modified to contain methyl groups. When proteins containing these methylated amino acids are degraded, free methylarginines are released into the cytosol and then enter the plasma. Symmetric dimethylarginine is eliminated primarily (≥ 90%) by renal clearance [4, 5]. Because serum SDMA is filtered by the kidneys, plasma concentrations are correlated with changes in glomerular filtration rate (GFR). The gold standard for estimating renal function is by measurement of GFR. A meta-analysis of 18 studies in humans showed that serum SDMA concentration is highly correlated with GFR [6]. In addition, we have shown that serum SDMA correlates with GFR in cats [3, 7] as well as in dogs [8]. Furthermore, serum SDMA concentrations are not affected by lean body mass in cats [7] or in dogs [9].

A review of the literature shows that increased SDMA is caused by reduced renal function, and by itself does not contribute to progression of renal disease (reviewed in [9]). Chronic SDMA infusion in otherwise healthy mice had no effect on renal function, renal histology, blood pressure, or cardiac function even though SDMA concentrations were increased an order of magnitude and comparable to those in patients with CKD [10]. In theory, SDMA could interfere with renal function by inhibiting L-arginine uptake, yet using the gold standard GFR measurement for mice (FITC-inulin clearance) researchers did not detect even minor changes. These data strengthen the role of SDMA as a marker of renal impairment that plays no pathophysiological role in of itself.

The use of a biomarker that correlates with deceasing GFR allows cats with CKD to be detected earlier in the disease course before azotemia develops, and may allow earlier treatment options that delay progressive loss of kidney function, while maintaining adequate nutrition. Currently, feeding a renal diet to cats with IRIS stage 2 CKD or higher is considered the standard of care with strong evidence supporting this recommendation [11]. Dietary modifications include decreased protein, phosphorus, and sodium content; increased water soluble vitamins and fiber content; increased caloric density; and additional n-3 fatty acids (FA), antioxidants, and potassium [11]. In one study, a renal diet was better when compared with an adult maintenance diet in minimizing uremic episodes and kidney-related deaths in cats with IRIS stage 2 or 3 CKD [1]. Now that serum SDMA concentrations can be used to identify cats with CKD earlier in the disease course, corresponding to IRIS stage 1 CKD, it is possible to perform clinical feeding trials with foods designed to promote healthy aging and stabilize renal function.

It is unknown whether dietary interventions in nonazotemic cats with increased serum SDMA (early renal insufficiency) will improve renal function, based on a decrease in serum SDMA concentrations. The purpose of this study was to evaluate the short term effects of a test food that contained functional lipids (fish oil), antioxidants (vitamins C and E), L-carnitine, botanicals (vegetables), high quality protein (wet meat chicken), and increased amino acids on circulating renal biomarkers and urinalysis results in healthy geriatric cats compared with cats fed owner’s-choice foods. The hypothesis of this study was that cats consuming test food would show improvement in GFR based on serum renal biomarker concentrations.

## Materials and Methods

### Cats and ethics statement

This study protocol was reviewed and approved by the Institutional Animal Care and Use Committee, Hill’s Pet Nutrition, Inc., Topeka, KS, USA (Permit Number: CP-523). Owners signed an informed consent form prior to enrollment of their cat, agreeing to comply with instructions given by their veterinarian and detailed in the consent form. Procedures were designed to avoid or minimize discomfort, distress, and pain. Cats were monitored for signs of disease. If an adverse event occurred, the cat’s health took precedence over continuation in the feeding trial.

### Study design

This was a prospective feeding study of 6-months duration. A contract research organization recruited eleven veterinary clinics from around the United States. Participating clinics were in Kansas, Oklahoma, California, New York, Pennsylvania, Missouri, Tennessee, Georgia, Colorado and Arizona. Cats were screened (physical examination, blood work, urinalysis) and selected by veterinarians to participate in the study. All cats remained with their owners throughout the study. Once cats were accepted into the study, they were randomized by gender to receive either test food or owner’s-choice foods. Cats within the same household were assigned to the same group, either both received test food or both received owner’s-choice foods. Cats were evaluated at baseline, and at 1, 2, 3 and 6 months. History, physical examination forms, medication records, dietary information, and both owner and veterinarian questionnaires were filled out electronically at each visit. Blood samples were obtained and submitted for analysis of selected serum analytes. Urine was collected to determine urine specific gravity (USG) and urine protein:Cr ratio, at baseline and at 3 and 6 months.

Inclusion criteria were that cats had to be ≥ 9 years old, housed indoors only, and have had ovariohysterectomy or been neutered, and were free of any chronic disease based on normal findings on physical examination, complete blood count, serum chemistry profile, complete urinalysis, total T4, and a negative FIV/FeLV tests. Exceptions included cats with mild arthritis, mild dental disease, or IRIS Stage 1 CKD [12]. IRIS Stage 1 CKD included cats with serum Cr < 1.6 mg/dL plus at least one other renal abnormality including abnormal kidneys on palpation or imaging, persistent proteinuria of renal origin, inadequate concentration of urine with no explanation other than renal origin, or concentrations of serum Cr that were progressively increasing. Cats also had to have a prior client-patient relationship with the attending veterinarian and a history of partaking in a good preventive medicine program, including routine vaccinations, deworming, and all recommended diagnostic testing performed. Up to two cats could be enrolled from one household.

Veterinarians and owners were compensated for their participation in the study. Study food was provided free of charge to pet owners participating in the study for the duration of the study. The veterinarians and the owners were blinded as to the sponsor of the study. The owners were instructed to maintain the test food at room temperature in the original food packaging. No special instructions were provided for handling foods of owner’s choice. If an owner asked, the response was that they should store the food as indicated according to the manufacturer’s instructions on the package.

Cats were excluded if they were receiving long-term systemic medication or had a history of chronic disease (e.g., heart disease; hyperthyroidism; inflammatory bowel disease; neoplasia; stage 2 to 4 CKD; diabetes mellitus; recurrent urinary tract disease including urolithiasis; pancreatitis; hepatic disease; severe dermatitis; and, severe periodontitis). Cats that belonged to a hospital employee of any hospital involved with the study were not eligible. Cats that were on a therapeutic or prescribed commercial brand pet food (other than a diet for weight loss or dental disease) or that were fed raw food were not eligible. Lastly, cats currently enrolled in another study were ineligible.

The criteria for removal of a cat from the study included failure to consume the study food, the owner requested to leave the study, if the veterinarian felt that the cat should not continue on the study, if there was poor owner compliance (e.g., routine administration of treats, dietary supplements, table scraps, neutraceuticals, or canned food to cats consuming the test food that was in excess of 10% of daily calories), if the cat had to be changed to a different food because a disease was diagnosed necessitating a change in food, if a life-threatening illness or accident occurred to the cat and the owner and the veterinarian determined that it was best not to continue the study, if the cat died, or if the owner elected euthanasia of the cat. If an adverse event occurred, a cat could continue the study if it was able to consume test food or, if in the control group, a non-therapeutic food. Also, if an adverse event occurred, and the cat was placed on chronic medication that adequately controlled a newly diagnosed condition (e.g., nonsteroidal anti-inflammatory drug for arthritis, insulin for diabetes) and the owner and veterinarian elected not to alter the cat’s food, a cat could continue the study.

There were a total of 105 cats initially enrolled in the study; mean age of 11.6 years (range 9 to 18 years). There were 39 males and 66 females. Mean initial body weight was 5.4 ± 1.5 kg (range 2.8–10.4 kg).

### Foods

The test food was produced by Hill’s Pet Nutrition, Inc., Topeka, KS, and met the nutritional requirements for adult cats (≥ 1 year) as established by the Association of American Feed Control Officials (AAFCO). Food was available in dry form only. Food composition, expressed as percentage of food, as fed, is shown in **Table 1**. Test food was a renal protective food containing functional lipids (fish oil), antioxidants (vitamins C and E), L-carnitine, botanicals (vegetables), and improved quality of protein sources (high bioavailability and an optimal amino acid composition). Test food contained 0.5% fish oil, additional α-tocopherol acetate (> 900 IU/kg, as fed), 200 mg/kg vitamin C, and 300 mg/kg L-carnitine. Test food also contained more biologically available protein sources than expected with commercially available owner’s-choice foods (wet meat chicken replaced other protein sources), and increased amino acid supplementation. In addition, there were increased amounts of vegetables: oat groats, beet pulp, tomato pomace, and broccoli powder.

**Table 1:** Food Composition of Test Food.

Nutrient^a^	Test Food
Moisture	8.17
Protein	32.94
Fat	15.82
Atwater Energy,^b^ kcal/kg	3,759
Ash	5.64
Crude Fiber	1.4
Calcium	0.80
Phosphorus	0.76
Sodium	0.46
Total tocopherols, IU/kg	909.0
Lauric acid [12:0]	0.01
Myristic acid [14:0]	0.12
Palmitic acid [16:0]	3.22
Stearic acid [18:0]	0.80
LA [18:2 (n-6)]	3.60
αLA [18:3 (n-3)]	0.22
ARA [20:4 (n-6)]	0.08
EPA [20:5 (n-3)]	0.11
DPA [22:5 (n-3)]	0.02
DHA [22:6 (n-3)]	0.07
SFA^c^	4.24
MUFA^d^	6.17
PUFA^e^	4.25
(n-6) FA^f^	3.75
(n-3) FA^g^	0.4
(n-6):(n-3) ratio	9.38

^a^ All analytical values are expressed as percentage of food, as fed, unless otherwise indicated.

^b^ Energy calculated using the modified Atwater factors as described [13].

^c^ Sum of the SFA: 8:0+10:0+11:0+12:0+14:0+15:0+16:0+17:0+18:0+20:0+22:0+24:0.

^d^ Sum of the MUFA: 14:1+15:1+16:1+17:1+18:1+20:1+22:1+24:1.

^e^ Sum of the PUFA: 18:2(n-6)+18:3(n-6)+18:3(n-3)+18:4(n-3)+20:2(n-6)+20:3(n-6)+20:3(n-3)+20:4(n-6)+20:4(n-3)+20:5(n-3)+21:5(n-3)+22:2(n-6)+22:4(n-6)+22:5(n-6)+22:5(n-3)+22:6(n-3).

^f^ Sum of the (n-6) fatty acids.

^g^ Sum of the (n-3) fatty acids.

Food analytical measurements and FA composition of the test food was determined by a commercial laboratory (Eurofins Scientific, Inc., Des Moines, IA) using Association of Analytical Communities (AOAC) methods. Test food FA were determined by gas chromatography of FA methyl esters. FA concentrations are expressed as g/100 g of FAs as fed. The sum of dietary SFA was determined as follows: 8:0+10:0+11:0+12:0+14:0+15:0+16:0+17:0+18:0+20:0+22:0+24:0. The sum of dietary MUFA was determined as follows: 14:1+15:1+16:1+17:1+18:1+20:1+22:1+24:1. The sum of dietary PUFA was determined as follows: 18:2(n-6)+18:3(n-6)+18:3(n-3)+18:4(n-3)+20:2(n-6)+20:3(n-6)+20:3(n-3)+20:4(n-6)+20:4(n-3)+20:5(n-3)+21:5(n-3)+22:2(n-6)+22:4(n-6)+22:5(n-6)+22:5(n-3)+22:6(n-3).

The test food was provided to the study sites by a commercial carrier, and each site received supplies of the test food both before and during the study period on an as needed basis. Pet owners were instructed to store the food in the package provided according to label instructions.

The owner’s-choice foods could be any dry, non-therapeutic, non-prescription food, excluding raw foods, of the owners choosing. Owners with cats in the control group were able to switch foods at their discretion.

Offering amounts were of equal calories based on the presumed resting energy requirement (RER) for each animal calculated by the formula: RER = 70 x (ideal body weight in kg) ^0.75^ with instructions to obtain and/or maintain ideal body weight.

### Serum and urine analyses

The same veterinary diagnostic laboratory (Marshfield Labs, Marshfield, WI) was used for determining blood hemoglobin, and serum sodium and renal function biomarker concentrations. Serum Cr and BUN concentrations were determined by enzymatic colorimetric methods. The reference intervals for blood hemoglobin (8.6 to 16.0 g/dL), serum sodium (146 to 160 mmol/L), serum Cr (0.6 to 2.0 mg/dL), and serum BUN (18.0 to 36.0 mg/dL) in adult cats were previously established. Serum SDMA concentrations were determined using liquid chromatography-mass spectrometry as previously described [3]. The reference interval for serum SDMA was determined by a commercial laboratory (IDEXX Laboratories, Westbrook, ME) in healthy cats with the upper 2.5% quartile being the upper limit of the reference range, i.e., < 14 μg/dL. All serum SDMA concentrations were determined retrospectively after the feeding trial ended from serum stored in serum banks.

Urine specific gravity was determined using a refractometer. Urine Cr concentration was used as an internal reference and measured with the same assay as serum Cr. Urine protein concentrations were determined using urine supernatant (benzethonium chloride turbidometric method). Urine protein to Cr (UPC) ratio calculations are reported as mg/dL protein: mg/dL Cr.

### Statistical analyses

Statistical analyses were performed using Statistical Analysis Software version 9.2 (SAS Institute, Cary, NC). Response variables were tested for normality by the Shapiro-Wilk test. To determine the effect of food, data from cats were analyzed as repeated-measures-in-time, randomized design using general linear models in PROC MIXED and the Satterthwaite approximation to determine the denominator degrees of freedom for the tests of fixed effects. Animal was considered the experiment unit. The variance-covariance structure of repeated measures of the same animal across time was modeled using an unstructured variance-covariance matrix, which was the most parsimonious model according to the Aikaike Information Criterion. Fixed effects in the model were treatment group (owner’s-choice foods, test food), time (0, 3, and 6 months), and their interaction. If there was a significant F-test, mean separation was completed by the PROC MIXED PDIFF statement. In a subset of cats that had a serum SDMA ≥14 μg/dL at baseline or during the 6-month feeding trial, a single measure t-test was performed to evaluate the effect of time on food. Data are reported as least square means (LSM) ± SEM. Statistical significance was declared at *P* ≤ 0.05.

## Results

A total of 80 cats completed the 6 month study. Eighteen of 51 cats (35.3%) consuming test food, and 7 of 54 cats (13.0%) consuming owner’s-choice foods had incomplete data or failed to complete the study. Of the 80 cats completing the study, mean age was 11.5 years (range, 9 to 18 years). There were 31 males and 49 females, with mean ± SD initial body weight of 5.4 ± 1.5 kg (range 2.8–9.5 kg) (**Table 2**).

**Table 2:** Cats completing the 6 month feeding trial were classified according to age, sex, initial body weight, and whether they were fed test food or owner’s-choice foods.

Food	N	Age, years Mean (range)	Sex	Initial Body Weight, kg Mean ± SD (range)
Test Food	33	11.7 (9–18)	20 SF; 13 NM	5.5 ± 1.5 (3.3–9.5)
Owner’s-Choice Foods	47	11.3 (9–17)	29 SF, 18 NM	5.3 ± 1.5 (2.8–9.1)

Concentrations of blood hemoglobin, and serum sodium and renal function biomarkers in cats at baseline, 3 months, and 6 months are shown in **Table 3** (least square means ± SEM**)**. Cats consuming test food showed significant decreases in serum Cr and BUN concentrations across time. Cats consuming owner’s-choice foods showed significant increases in serum SDMA concentrations at 3 and 6 months compared with baseline (all *P* ≤ 0.05). For serum SDMA (*P* = 0.03), Cr (*P* = 0.02), and BUN (*P* = 0.001), there were significant interactions between diet and time on study.

**Table 3:** Hemoglobin, serum sodium and renal function biomarkers, and urinalysis parameters of cats at baseline (initial) and after consuming test food or owner’s-choice foods for 3 and 6 months (mean ± SEM).

	Owner’s- Choice Foods	Test Food	Two-way ANOVA Analysis^†^ (*P* values)		
Number of Animals, N	47	33	Main Effect Diet	Main Effect Time	Effect of Diet x Time
**Hemoglobin:**					
*Hemoglobin (g/dL)*			0.88	0.27	0.30
Initial	13.44 ± 0.21	13.17 ± 0.26			
3 months	13.32 ± 0.25	13.41 ± 0.30			
6 months	13.53 ± 0.34	13.88 ± 0.42			
**Serum electrolytes:**					
*Serum Sodium (mmol/L)*			0.42	0.02	0.02
Initial	152.3 ± 0.31^a^	151.9 ± 0.38			
3 months	151.1 ± 0.27^b^	151.4 ± 0.32			
6 months	150.9 ± 0.29^b^	151.9 ± 0.35			
**Renal function markers**:					
*Urea Nitrogen (mg/dL)*			0.21	0.001	0.001
Initial	27.96 ± 0.86	28.62 ± 1.04^a^			
3 months	28.15 ± 0.89	25.52 ± 1.06^b^			
6 months	27.99 ± 0.81	25.35 ± 0.96^b^			
*Serum Creatinine (mg/dL)*			0.03	0.002	0.02
Initial	1.45 ± 0.04	1.41 ± 0.05^a^			
3 months	1.44 ± 0.04	1.26 ± 0.05^b^			
6 months	1.42 ± 0.04	1.23 ± 0.05^b^			
*Serum SDMA (μg/dL)*			0.61	0.17	0.03
Initial	10.32 ± 0.43^a^	10.29 ± 0.52			
3 months	11.19 ± 0.53^b^	9.98 ± 0.63			
6 months	11.39 ± 0.53^b^	9.74 ± 0.63			
**Urinalysis parameters:**					
*Urine Specific Gravity*			0.02	0.26	0.41
Initial	1.043 ± 0.002^a^	1.044 ± 0.002			
3 months	1.039 ± 0.002^b^	1.043 ± 0.002			
6 months	1.038 ± 0.002^b^	1.042 ± 0.002			
*Urine Protein*:*Cr Ratio*			0.41	0.88	0.18
Initial	0.12 ± 0.02	0.18 ± 0.02			
3 months	0.18 ± 0.04	0.17 ± 0.04			
6 months	0.23 ± 0.06	0.17 ± 0.07			

^†^*P* values are shown for diet main effects, time main effects, and for interaction of diet and time.

^a,b^ Means with different superscripts within a column are different between times at *P*≤0.05.

Hemoglobin concentrations remained stable across time for cats consuming both test food and owner’s-choice foods. Serum sodium concentrations decreased across time in cats consuming owner’s-choice foods at 3 and 6 months compared with baseline (*P* ≤ 0.05), but were stable in cats consuming test food. For serum sodium (*P* = 0.02), there was a significant interaction between diet and time on study.

Urine specific gravity in cats consuming owner’s-choice foods also showed significant decreases at 3 and 6 months compared with baseline (all *P* ≤ 0.05). There was no change in either group of cats for urine protein:Cr ratio across time.

A subset of nonazotemic cats with increased serum SDMA concentrations (indicating early renal insufficiency) was also analyzed. At baseline or during the 6-month feeding trial, 23/80 (28.8%) cats had serum SDMA ≥14 μg/dL and serum Cr <2.0 mg/dL (**Tables 4 and 5**). This included 17 cats fed owner’s-choice foods and six cats fed test food. Based on serum SDMA concentrations, of the 17 cats fed owner’s-choice foods, three improved during the course of the study (serum SDMA decreased), 13 got worse (serum SDMA increased), and one stayed the same (no change in serum SDMA). The increase in serum SDMA concentration was significant (*P* = 0.02) for cats fed owner’s-choice foods. Of the six cats fed test food, three improved (serum SDMA decreased), two got worse (serum SDMA increased), and one stayed the same (no change in serum SDMA).

**Table 4:** Hemoglobin, serum sodium and renal function biomarkers, and urinalysis parameters in a subset of cats that had serum SDMA concentrations ≥14 μg/dL (indicating renal insufficiency) at baseline or during the 6-month feeding trial.

	Owner’s-Choice Foods	Test Food
**Number of Animals, N**	17	6
**Hemoglobin:^a^^,^^b^**		
*Hemoglobin (g/dL)*		
Initial	13.23 ± 0.21	14.00 ± 0.55
3 months	12.86 ± 0.26	14.00 ± 0.60
6 months	12.74 ± 0.34	13.47 ± 1.10
Change	-0.49 ± 1.01	-0.53 ± 0.61
P value	*P* = 0.60	*P* = 0.68
**Serum electrolytes:**		
*Serum Sodium (mmol/L)*		
Initial	152.1 ± 0.53	153.0 ± 0.90
3 months	151.1 ± 0.58	152.3 ± 0.95
6 months	151.1 ± 0.64	152.3 ± 1.08
Change	-1.1 ± 0.49	-0.7 ± 0.83
P value	*P* = 0.03	*P* = 0.43
**Renal function markers:**		
*Urea Nitrogen (mg/dL)*		
Initial	30.47 ± 1.66	28.67 ± 2.80
3 months	29.13 ± 1.71	27.33 ± 2.80
6 months	29.71 ± 1.66	26.33 ± 2.80
Change	-0.76 ± 1.43	-2.33 ± 1.28
P value	*P* = 0.60	*P* = 0.13
*Serum Creatinine (mg/dL)*		
Initial	1.59 ± 0.08	1.50 ± 0.13
3 months	1.64 ± 0.08	1.47 ± 0.13
6 months	1.52 ± 0.08	1.48 ± 0.13
Change	-0.06 ± 0.09	-0.02 ± 0.13
P value	*P* = 0.47	*P* = 0.90
*Serum SDMA (μg/dL)*		
Initial	12.32 ± 0.92	14.19 ± 1.55
3 months	15.06 ± 0.95	13.15 ± 1.55
6 months	14.47 ± 0.92	14.42 ± 1.55
Change	2.16 ± 0.81	0.23 ± 1.46
P value	*P* = 0.02	*P* = 0.88
**Urinalysis parameters:**		
*Urine Specific Gravity*		
Initial	1.040 ± 0.004	1.035 ± 0.007
3 months	1.034 ± 0.004	1.033 ± 0.007
6 months	1.033 ± 0.004	1.032 ± 0.007
Change	-0.006 ± 0.003	-0.004 ± 0.003
P value	*P* = 0.07	*P* = 0.28
*Urine Protein*:*Cr Ratio*		
Initial	0.12 ± 0.12	0.15 ± 0.20
3 months	0.23 ± 0.13	0.17 ± 0.20
6 months	0.36 ± 0.13	0.20 ± 0.20
Change	0.24 ± 0.22	0.05 ± 0.04
P value	*P* = 0.29	*P* = 0.26

^a^Shown are values at baseline (initial) and after consuming test food or owner’s-choice foods for 3 and 6 months (mean ± SEM).

^b^*P* values are shown for change over time within diet.

**Table 5:** Serum SDMA and creatinine biomarker concentrations, and urine specific gravity in individual cats that had serum SDMA concentrations ≥14 μg/dL (indicating renal insufficiency) at baseline or during the 6-month feeding trial.

Name, time on food	Age (y)	Sex	Serum SDMA (μg/dL)	Serum Creatinine (mg/dL)	Urine Specific Gravity	Overall Response^b^
**Cats fed owner’s-choice foods^a^**						
*Katlee*	10.5	SF				
Initial			11.8	1.1	1.044	
3 months			11.6	1.2	1.036	
6 months			17.1	1.1	1.038	Worse
*Ashley*	10.5	SF				
Initial			11.0	1.2	1.065	
3 months			12.9	1.2	1.054	
6 months			18.3	1.3	1.026	Worse
*Harvick*	10	NM				
Initial			12.5	1.7	1.057	
3 months			17.7	1.5	1.047	
6 months			17.3	1.5	1.042	Worse
*Fred*	16	NM				
Initial			8.6	1.5	1.054	
3 months			10.6	2.4	1.018	
6 months			15.4	1.9	1.027	Worse
*Ophelia*	14.5	SF				
Initial			10.0	1.4	1.056	
3 months			14.5	1.4	1.039	
6 months			15.7	1.1	1.047	Worse
*Ellie*	11.5	SF				
Initial			13.3	1.7	1.017	
3 months			17.8	1.9	1.014	
6 months			14.2	2.3	1.015	Worse
*Ben*	12	NM				
Initial			8.9	1.6	1.012	
3 months			16.7	1.9	1.015	
6 months			11.1	2.0	1.013	Worse
*Jerry*	12	NM				
Initial			7.1	1.1	1.035	
3 months			14.5	1.4	1.028	
6 months			8.9	1.3	1.022	Worse
*Newman*	14.5	NM				
Initial			15.3	1.8	1.050	
3 months			16.5	1.6	1.052	
6 months			14.2	1.5	1.062	Improved
*Ginger*	14	SF				
Initial			20.1	1.9	1.019	
3 months			NA	NA	NA	
6 months			16.2	1.5	1.015	Improved
*Tiger*	13.5	NM				
Initial			8.7	1.4	1.040	
3 months			14.1	1.5	1.027	
6 months			11.0	1.4	NA	Worse
*Thomas*	10.5	NM				
Initial			10.9	1.4	1.050	
3 months			14.0	1.4	1.053	
6 months			10.4	1.0	1.050	Stable
*Heather*	10.5	SF				
Initial			13.8	1.6	1.049	
3 months			15.3	1.7	1.045	
6 months			15.3	1.4	1.051	Worse
*Cleo*	15.5	SF				
Initial			22.9	1.7	1.013	
3 months			26.5	1.5	1.013	
6 months			25.4	1.4	1.015	Worse
*Ella*	13.5	SF				
Initial			9.8	2.4	1.047	
3 months			15.0	2.1	NA	
6 months			12.9	1.7	1.047	Worse
*Olive*	13	SF				
Initial			10.5	1.6	1.020	
3 months			14.1	1.9	1.018	
6 months			12.8	1.9	1.022	Worse
*Shadow*	9	SF				
Initial			14.3	1.9	1.058	
3 months			9.1	1.7	1.051	
6 months			9.9	1.5	NA	Improved
**Cats fed test food**:						
*Sasha*	9.5	SF				
Initial			18.2	1.5	1.054	
3 months			18.3	1.9	1.047	
6 months			15.8	1.6	1.047	Improved
*Taffy*	11.5	SF				
Initial			14.3	1.3	1.025	
3 months			8.9	1.1	1.032	
6 months			10.4	0.9	1.033	Improved
*Missy*	15	SF				
Initial			14.1	1.6	1.026	
3 months			16.0	1.8	1.017	
6 months			18.5	2.1	1.015	Worse
*Angela*	15	SF				
Initial			10.0	1.4	1.030	
3 months			9.4	1.4	1.033	
6 months			14.7	1.4	1.031	Worse
*Terp*	12	SF				
Initial			13.4	1.6	1.021	
3 months			14.3	1.3	1.015	
6 months			13.4	1.3	1.019	Stable
*Ozzy*	11	NM				
Initial			15.2	1.6	1.056	
3 months			12.0	1.3	1.053	
6 months			13.7	1.6	1.045	Improved

^a^Shown are values at baseline (initial) and after consuming owner’s-choice or test foods for 3 and 6 months.

^b^Based on change in serum SDMA concentration over 6 month feeding period.

## Discussion

Glomerular filtration rate is directly related to functional renal mass. We have previously shown in cats that serum SDMA concentrations are inversely related to GFR [3, 7], that serum SDMA concentrations can be used to detect renal dysfunction earlier in cats with chronic renal disease compared with serum Cr concentrations [3], and that serum SDMA concentrations are not affected by lean body mass [7]. The upper reference interval for serum SDMA (< 14 μg/dL) corresponds to a reduction in GFR of approximately 24% from mean GFR, whereas the upper reference interval for serum Cr corresponds to a reduction of approximately 60% from mean GFR in cats [3]. On average, serum SDMA detects a reduction in GFR 17.0 months before serum Cr in cats with chronic renal disease [3].

The purpose of this study was to determine if dietary interventions in nonazotemic cats with increased serum SDMA concentrations (early renal insufficiency) could improve renal function based on a decrease in serum SDMA. Our results suggest that cats with early renal insufficiency fed a test food designed to promote healthy aging, over a 6 month period, were more likely to have stable renal function evidenced by stable serum SDMA concentrations compared with cats fed owner’s-choice foods. Cats fed owner’s-choice foods were more likely to have progressive renal insufficiency characterized by increasing serum SDMA concentrations.

Because abnormal serum sodium concentrations are common in human CKD patients, both hypo- and hypernatremia, their risk increases with advancing stage of CKD, and they are associated with a significant increase in mortality [14], we also assessed serum sodium concentrations in cats overall, as well as in the subset of nonazotemic cats with increased serum SDMA concentrations (indicating early renal insufficiency). Serum sodium concentrations decreased significantly across time in cats consuming owner’s-choice foods, but were stable in cats consuming test food. Changes may be biologically insignificant as concentrations remained within the reference interval. Hemoglobin concentrations also remained stable across time for cats consuming both test food and owner’s-choice foods.

Test food was energy-dense and contained functional lipids (fish oil), antioxidants (vitamins C and E), L-carnitine, botanicals (vegetables), high quality protein (wet meat chicken), and increased amino acids. Traditional nutritional studies have focused on individual nutrients or foods, but their additive or interactive influences are more apparent when complete diets or several nutritional interventions in combination are studied in healthy aging trials [15]. In humans, the decline in renal function that occurs in a large percentage of aging and CKD populations is likely linked to increased levels of oxidative stress and inflammation [16]. Food is a major source of oxidants, and diets can be modified to decrease oxidant burden [16]. In this study, feeding test food for 6 months reversed the increase in serum SDMA concentration in three of six cats, and serum SDMA concentration remained stable in one cat.

Several clinical trials support using renal diets to delay the onset of uremia and premature death from CKD complications (reviewed in [11, 17, 18]). Previous recommendations were that nutritional management should begin when cats developed azotemia, i.e., with IRIS stage 2 and higher CKD [1, 19]. This study shows there is benefit to initiating dietary therapy earlier, i.e., with IRIS stage 1 CKD in order to slow progression of CDK.

Renal diets are modified from maintenance diets in several ways including supplementation with (n-3) polyunsaturated fatty acids and the addition of antioxidants. A proinflammatory diet, based on the assumed proinflammatory effects of certain nutrients, vitamins and trace elements, is associated with systemic inflammation as well as with reduced kidney function [20]. Thus, inflammation may be one of the pathways through which diet can affect kidney function [20]. The rationale for feeding dietary fish oil, is that eicosapentaenoic acid (EPA) and docosahexaenoic acid (DHA) from fish oil influence the physical nature of cell membranes and membrane protein-mediated responses, lipid-mediator generation, cell signaling, and gene expression in many different cell types [21]. In particular, eicosanoids derived from EPA may protect against excessive inflammatory reactions, whereas eicosanoids derived from arachidonic acid may exacerbate inflammatory reactions. In dogs, it has been shown that the rate of decline of GFR is slowed by the use of (n-3) polyunsaturated fatty acids and by the addition of dietary antioxidants [22–24].

L-carnitine supplementation also has been shown to decrease markers of oxidative stress and inflammation in patients with chronic diseases, including CKD, as reviewed in [25]. For example, evidence shows that carnitine prevents oxidative stress and inflammation by inhibiting production of reactive oxygen species and inflammatory cytokines. A recent meta-analysis supports a clinical benefit of L-carnitine supplementation in lowering circulating levels of C-reactive protein [26].

Oral supplementation with vitamins C and E, in combination [27, 28], or as a micronutrient cocktail containing physiologic doses of antioxidant vitamins and trace minerals [28], can decrease oxidative stress in humans. In human CKD patients, oxidative stress is common and considered to be an important pathogenic mechanism [29]. The majority of studies investigating anti-oxidant treatments in CKD patients show a reduction in oxidative stress and many show improved renal function (reviewed in [29, 30]). Thus, diet may affect kidney function by altering the balance between antioxidants and oxidizing species.

In conclusion, 28.8% of client-owned geriatric cats with early stage kidney disease, consistent with IRIS Stage 1 CKD, were identified with increased serum SDMA and normal serum Cr concentrations over a 6-month period. Those cats that were switched to a test food that contained functional lipids (fish oil), antioxidants (vitamins C and E), L-carnitine, botanicals (as vegetables), high quality protein (wet meat chicken), and increased amino acids were more likely to maintain their serum SDMA concentration than cats that continued to consume foods of owner’s-choice. These results suggest that nonazotemic cats with elevated serum SDMA (early renal insufficiency) fed a food designed to promote healthy aging are more likely to demonstrate stable renal function compared with cats fed owner’s-choice foods. Cats fed owner’s-choice foods are more likely to demonstrate progressive renal insufficiency.
